# Genome-Wide Characterization of Genetic Diversity and Population Structure in a Kazakhstani Two-Row Spring Barley Breeding Panel

**DOI:** 10.3390/ijms27167466

**Published:** 2026-08-20

**Authors:** Yuliya Genievskaya, Vladimir Chudinov, Grigoriy Sereda, Laura Tokhetova, Saule Abugalieva, Yerlan Turuspekov

**Affiliations:** 1 Institute of Plant Biology and Biotechnology, Almaty 050040, Kazakhstan; julia.genievskaya@gmail.com (Y.G.); absaule@yahoo.com (S.A.); 2 Karabalyk Agricultural Experimental Station, Nauchnoe village 110908, Kazakhstan; ch.den@mail.ru; 3 A.F. Khristenko Karaganda Agricultural Experimental Station, Tsentralnoe village 100435, Kazakhstan; sereda_t@bk.ru; 4 I. Zakhayev Kazakh Research Institute of Rice Growing, Kyzylorda 120008, Kazakhstan; lauramarat_777@mail.ru

**Keywords:** breeding, genetic diversity, *Hordeum vulgare* L., phenotypic differentiation, population stratification

## Abstract

Barley (*Hordeum vulgare* L.) is a major cereal in Kazakhstan, where diverse breeding material supports crop improvement. We characterized 86 two-row spring barley accessions from six breeding organizations using the Illumina Infinium 50K Barley SNP Array. Analysis of 29,920 high-quality SNPs revealed moderate diversity (*H_e_* = 0.346, PIC = 0.278, Shannon = 0.752), with 80.84% of molecular variation occurring within and 19.16% among breeding organizations. A minor allele frequency-free (MAF-free) analysis showed that 95.61% of marker–allele combinations at polymorphic loci were shared by at least two organizations. Although PCA, kinship, and neighbor-joining analyses indicated extensive overlap, discriminant analysis of principal components (DAPC) cluster membership was significantly associated with breeding origin (χ^2^ = 106.38, Monte Carlo *p* = 1 × 10^–5^; bias-corrected Cramér’s *V* = 0.513), demonstrating substantial but incomplete differentiation among breeding programs. Phenotypic differentiation was evaluated using environment-adjusted genotype BLUPs. All seven traits differed significantly among five DAPC clusters. In a reduced six-trait linear discriminant analysis (LDA) excluding vegetation period, LD1 was associated most strongly with number of kernels per spike, followed by heading time, spike length, and heading-to-maturity time. Leave-one-out cross-validation (LOOCV) accuracy was 36.47%, exceeding the permutation mean of 19.83% but indicating considerable phenotypic overlap. The examined materials therefore constitute a diverse, interconnected breeding panel that may support germplasm management and parent selection and provide a genomic and phenotypic framework for future GWAS, genomic selection, and targeted validation of molecular markers within the represented collections.

## 1. Introduction

Cereals constitute the foundation of global crop production and play a central role in ensuring food security, livestock production, and industrial raw materials. According to the Food and Agriculture Organization (FAO) [1], cereal crops occupy more than 740 million hectares worldwide, representing the largest share of harvested cropland and nearly twice the area devoted to the next largest crop groups, including coarse grains and oil crops. Among cereals, barley (*Hordeum vulgare* L.) ranks as the fourth most important crop after maize, wheat, and rice [1]. Owing to its broad ecological adaptability and tolerance to drought, salinity, and low temperatures, barley is cultivated across diverse agroecological environments and is widely used as livestock feed, malted, brewed, and consumed by humans [2].

Kazakhstan is one of the world’s major barley producers, ranking fifth globally according to recent USDA statistics [3]. Within the country, barley is the second most important cereal crop after wheat, although its production is substantially lower. In 2025, Kazakhstan harvested 19.33 million tons of wheat compared with 3.59 million tons of barley [4]. The crop is cultivated predominantly for feed production and represents an essential component of the country’s livestock industry, while malting barley is also important for the brewing sector. Despite its strategic importance, the average national barley yield reached only 1.67 t/ha in 2025, considerably below the average yields reported for the European Union (5.63 t/ha), Australia (3.48 t/ha), and Russia (3.03 t/ha) [3]. Improving the genetic potential of modern barley breeding germplasm is therefore essential for increasing productivity under the diverse and often harsh environmental conditions of Kazakhstan.

The development of improved barley cultivars relies on the availability and effective utilization of genetic diversity within breeding germplasm. Although modern breeding has substantially enhanced agronomic performance, continuous selection for elite cultivars has simultaneously reduced the genetic base of breeding materials, emphasizing the importance of identifying and incorporating novel allelic variation from breeding collections, landraces, and wild relatives [5,6]. Consequently, comprehensive characterization of genetic diversity has become essential for broadening the breeding pool, improving adaptation, and increasing resilience to emerging biotic and abiotic stresses. This has been demonstrated by large international collections such as the USDA Barley Core Collection, where genome-wide SNP genotyping uncovered considerable untapped allelic diversity directly relevant to breeding and trait discovery [7]. Beyond estimating overall diversity, population structure analysis provides insight into the genetic relationships among breeding materials by identifying genetic groups, quantifying admixture, and assessing genetic differentiation. Such information supports germplasm management, facilitates the selection of genetically divergent parental lines, and is indispensable for genome-wide association studies (GWAS), where correction for population stratification minimizes false-positive marker–trait associations [8,9,10].

Over the past two decades, a wide range of molecular marker systems has been employed to investigate genetic diversity and population structure in barley, including amplified fragment length polymorphisms (AFLPs), simple sequence repeats (SSRs), Diversity Arrays Technology (DArT), and, more recently, genome-wide single-nucleotide polymorphism (SNP) markers. The introduction of high-throughput SNP genotyping platforms, including the Illumina 9K Infinium iSelect array [11] and the barley 50K Illumina Infinium SNP array [12], has greatly increased the resolution of diversity studies. Owing to their high marker density, reproducibility, and genome-wide coverage, genome-wide SNP markers have become the standard approach for characterizing genetic diversity and population structure in barley.

A worldwide survey of cultivated barley germplasm spanning Europe, the Americas, Asia, and Africa, including material from the former Soviet republics, demonstrated that population structure is shaped jointly by geographic origin, end-use, and breeding history [13]. A parallel study of a highly structured Mediterranean-basin association panel of 192 accessions used clustering and principal coordinate analysis to resolve five distinct groups centered on key ancestors and regions of origin, underscoring the strong geographic signal retained in cultivated germplasm even after decades of breeding [14]. More recently, genomic characterization of the global IPK Gatersleben collection (over 21,000 accessions) confirmed this pattern at a genome-wide scale, with principal component analysis separating eastern from western barley and further isolating Ethiopian material as a distinct group whose genomic profile closely tracked geographic collection site [5].

Studies focused on the Near Eastern center of origin have reinforced the link between population structure and domestication history. In a collection of 144 Iranian landraces and breeding lines, spike row type (two-rowed versus six-rowed) emerged as the dominant factor driving clustering at *K* = 2, and allelic distribution correlated with domestication history and eco-geographic factors, with Iranian landraces retaining substantially higher diversity than introduced material [15]. European regional collections show a comparable pattern shaped instead by recent breeding history: a Serbian barley collection spanning a 40-year breeding period separated into three structural groups, with coordinate analysis grouping genotypes specifically by growth habit and row type [16], while Polish spring landraces collected during late-20th-century genebank expeditions retained considerable genetic variability of clear value for future breeding programs [17].

In Asia, studies of highland barley on the Qinghai-Tibetan Plateau illustrate how population structure can additionally capture local adaptation: genomic analysis of 1308 highland and 58 inland Chinese landraces resolved six subpopulations that clearly separated the six-rowed, naked “Qingke” barley of Tibet from inland barley, revealing multiple origins of Qingke and a correspondence between subpopulation distribution and specific ecological environments [18]. In India, structural analysis of wild barley germplasm evaluated for resistance to corn leaf aphid unanimously resolved three subpopulations using multivariate, Bayesian, and PCoA methods, linking genetic structure to a biotic stress phenotype [19]. North American breeding germplasm displays yet another organizing axis: analysis of 3840 elite US barley lines identified nine subpopulations, largely reflecting row type, end-use (malting versus feed), and growth habit, consistent with earlier structure delineated in this germplasm pool [20]. In Africa, characterization of Ethiopian barley accessions using SSR markers revealed moderate genetic differentiation correlated with geographic origin, highlighting how incomplete characterization of this germplasm continues to limit targeted breeding and conservation efforts [21].

Central Asian, and specifically Kazakhstani, barley germplasm has only recently been incorporated into this global picture, despite Kazakhstan’s importance as a barley producer. Genome-wide SNP analysis of wild barley populations from southern Kazakhstan found narrower genetic diversity (*H_e_* = 0.19) relative to Middle Eastern wild germplasm (*H_e_* = 0.29), consistent with a comparatively recent, founder-limited spread of wild populations adapted to low winter temperatures [22]. Building on this, a study of two-rowed cultivated barley from Kazakhstan showed that domestic accessions form a distinct subcluster most closely related to European germplasm when compared against six world regions, while finer-scale analysis of six domestic breeding programs resolved five subclusters and identified 214 SNPs with divergent allele frequencies linked to genes underlying adaptive traits such as heading date and plant height [23]. More recently, Genievskaya et al. (2024) [24] demonstrated the importance of accounting for population structure during genome-wide association analysis of Kazakh breeding material. Complementing these SNP-based works, an SSR-based characterization of a Kazakhstani two-rowed spring barley collection combined neighbor-joining clustering, PCoA, and STRUCTURE analysis to resolve population structure and identify SSR alleles associated with resistance to barley scald and net blotch, linking diversity assessment directly to disease-resistance breeding priorities in the region [25]. In contrast to barley, the genetic diversity and population structure of Kazakh wheat germplasm have been investigated much more extensively using both genome-wide SNP markers and association mapping approaches, providing detailed insights into the genetic organization of breeding collections and the distribution of adaptive alleles [26,27].

Collectively, previous studies demonstrate that barley population structure is consistently shaped by factors such as spike row type, growth habit, geographic origin, end-use, and breeding history. Therefore, the present study aimed to characterize genome-wide genetic diversity and population structure within a defined panel of 86 two-row spring barley accessions representing six breeding organizations in Kazakhstan. The panel comprised officially registered cultivars, cultivars not registered for use in Kazakhstan, and advanced breeding lines maintained by the participating organizations. Specifically, we aimed to (i) characterize genome-wide genetic diversity within and among breeding groups; (ii) investigate population structure and genetic relationships among breeding materials; (iii) quantify genetic differentiation among accessions representing six breeding organizations; (iv) determine whether the inferred genetic clusters were associated with breeding origin; and (v) evaluate phenotypic differentiation among the SNP-derived genetic clusters and identify the agronomic traits contributing most strongly to this differentiation.

## 2. Results

### 2.1. Genome-Wide SNP Variation and Genetic Diversity

**Figure 1:**
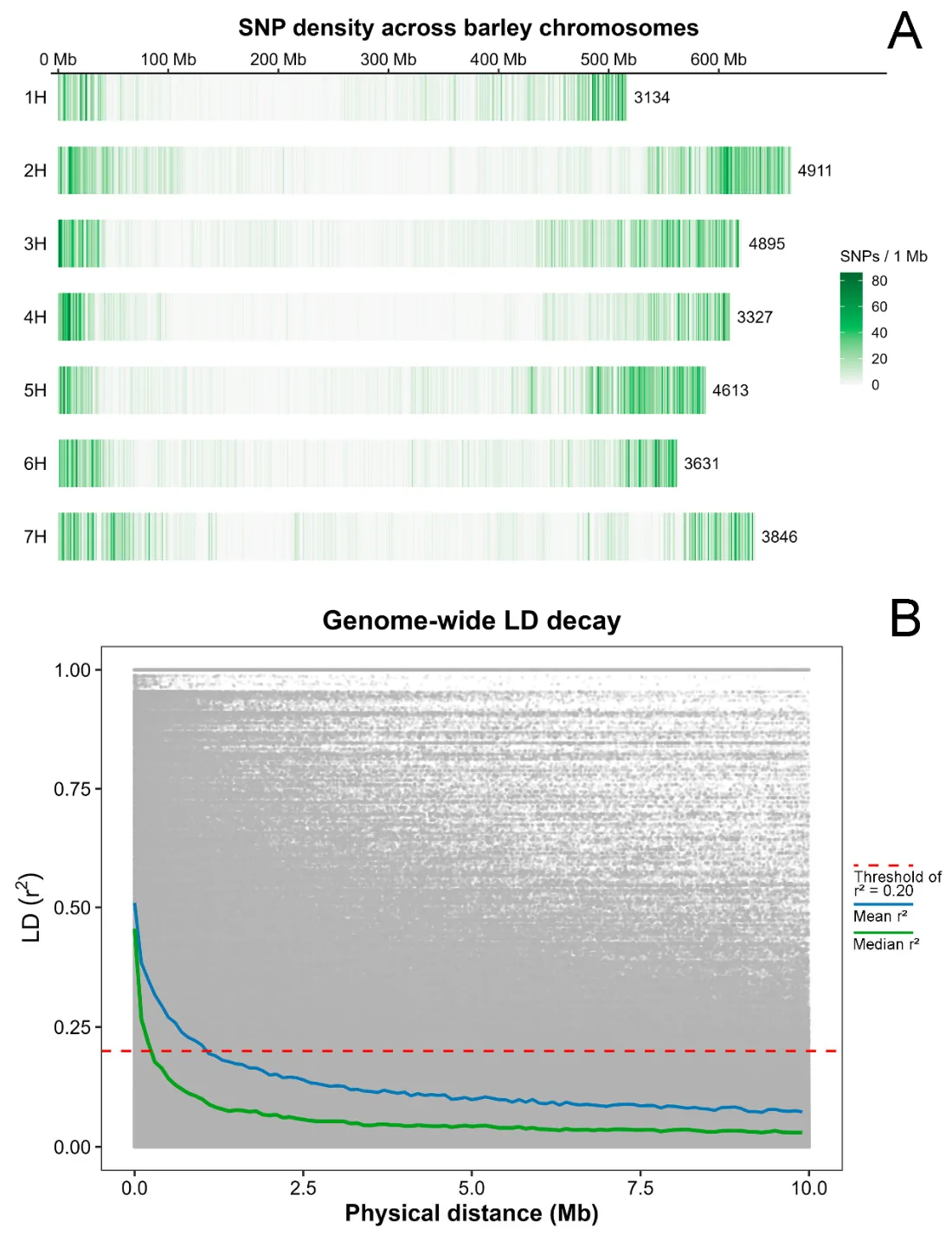
Genome-wide SNP density and linkage disequilibrium (LD) decay in the barley panel. (**A**) Chromosomal distribution and density of 28,357 SNPs with known physical positions across the seven barley chromosomes (1H–7H) calculated within 1 Mb windows. (**B**) Genome-wide LD decay plot based on 86 barley accessions.

Genotyping of 86 barley accessions representing six Kazakh agricultural research organizations yielded 44,040 SNP markers. Following quality filtering, 29,920 high-quality SNPs were retained for genetic diversity and population structure analyses. Of these, 28,357 SNPs had known physical positions on the Morex v3 reference genome and were used for chromosome-based analyses, including SNP density and linkage disequilibrium (LD) (Figure 1). The complete genotyping matrix and the corresponding SNP information file have been deposited in the Zenodo repository and are publicly available [28].

The number of mapped SNPs ranged from 3134 on chromosome 1H to 4911 on chromosome 2H, followed by 4895 on chromosome 3H, 4613 on chromosome 5H, 3846 on chromosome 7H, 3631 on chromosome 6H, and 3327 on chromosome 4H. Marker density was unevenly distributed along all chromosomes, with regions of high and low SNP density observed throughout the genome (Figure 1A). Genome-wide LD decreased with increasing physical distance between markers (Figure 1B). Using the threshold of r2 = 0.20, the mean genome-wide LD decayed over a distance of 1.068 Mb, whereas the corresponding median LD decay was 0.244 Mb. Comparable LD decay patterns were observed across all seven barley chromosomes (Figure S1), although decay distances differed among chromosomes. Mean LD decay ranged from 0.749 Mb (4H) to 1.272 Mb (3H), while median LD decay ranged from 0.131 Mb (7H) to 0.409 Mb (2H). Genetic diversity indices calculated for the six breeding groups of barley are summarized in Table 1.

Mean SNP call rate was consistently high across all populations, ranging from 99.70 ± 2.99% in Krasnovodopad Agricultural Experimental Station (KvAES) to 99.89 ± 1.01% in Kazakh Research Institute of Rice Growing (KRIRG), with an overall mean of 99.84 ± 1.57%. Mean minor allele frequency (MAF) varied between 0.20 ± 0.14 in Kazakh Research Institute of Agriculture and Plant Growing (KRIAPG) and 0.24 ± 0.14 in Karabalyk Agricultural Experimental Station (KbAES), while the complete barley panel showed an overall mean MAF of 0.25 ± 0.15.

Expected heterozygosity (*H_e_*) ranged from 0.272 ± 0.188 in Karaganda Agricultural Experimental Station (KaAES) to 0.328 ± 0.149 in KbAES, with an overall value of 0.346 ± 0.169. Similarly, polymorphism information content (PIC) ranged from 0.217 ± 0.143 to 0.263 ± 0.109, whereas the total collection reached 0.278 ± 0.127. Shannon’s information index varied from 0.586 ± 0.376 in KaAES to 0.709 ± 0.281 in KbAES, with an overall value of 0.752 ± 0.333. Observed heterozygosity (*H_o_*) was consistently low across all breeding groups, ranging from 0.0003 ± 0.0053 to 0.0009 ± 0.0162, whereas the inbreeding coefficient (*F_IS_*) remained uniformly high (0.996–0.998) in all populations and reached 0.999 ± 0.035 in the complete collection. The proportion of polymorphic loci (PPL) ranged from 73.10% in KvAES to 93.14% in KbAES.

Reanalysis of the call-rate-filtered dataset without a MAF threshold retained all 86 accessions and 39,952 SNPs, of which 36,156 were polymorphic. A total of 3177 private alleles were identified, including 1049 supported by at least two accessions (Table 2).

Private alleles represented 4.39% of the observed marker–allele combinations at polymorphic loci, whereas 95.61% were shared by at least two breeding organizations. After rarefaction to six accessions per organization, mean private-allele richness ranged from 79.4 in KaAES to 882.2 in KbAES, indicating a shared core allelic pool accompanied by pronounced organization-specific enrichment, particularly in KbAES (Table 2). Detailed results on genetic diversity statistics for individual barley accessions and SNP markers, chromosome-specific LD, and private SNPs are presented in File S1.

### 2.2. Genetic Differentiation Among Breeding Groups

To further evaluate relationships among six barley breeding groups, analysis of molecular variance (AMOVA) was performed. The among-group component accounted for 19.16% of the total molecular variation, whereas the remaining 80.84% was attributed to variation within breeding groups (*p* = 0.001) (Table 3).

**Figure 2:**
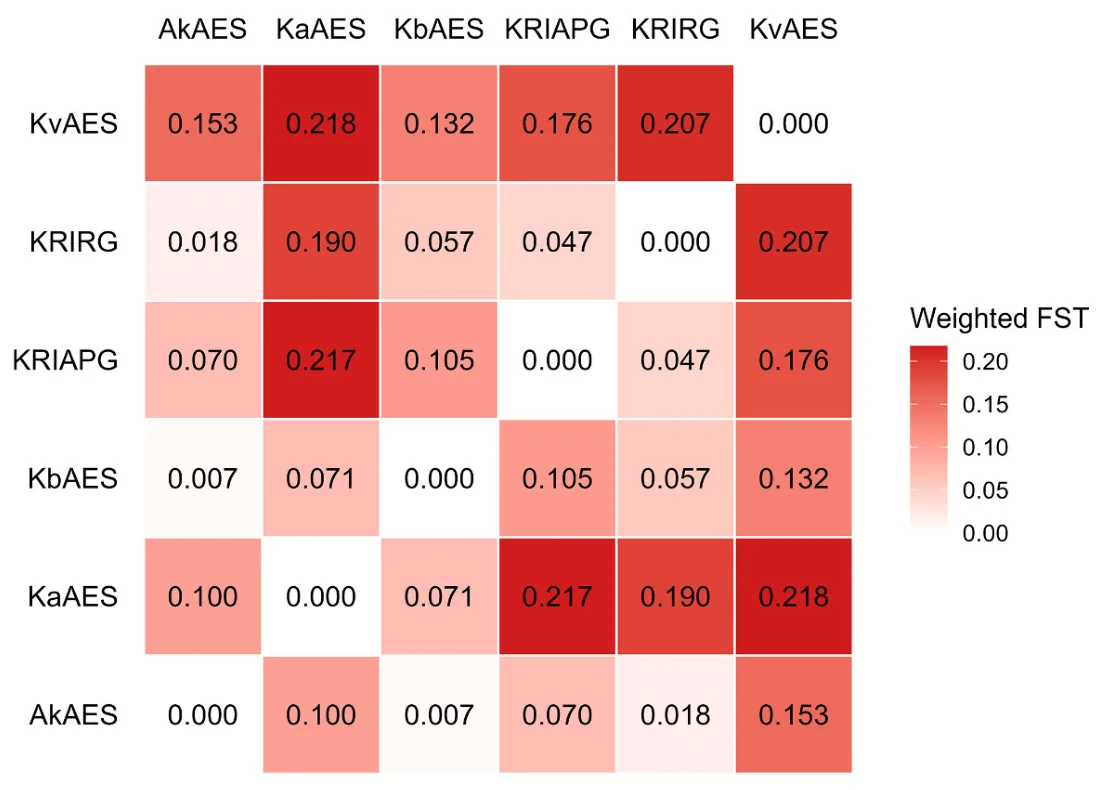
Pairwise weighted *F_ST_* matrix among six Kazakh barley breeding groups of origin.

Pairwise genetic differentiation among breeding groups was further evaluated using weighted *F_ST_* estimates (Figure 2).

Pairwise *F_ST_* values ranged from 0.007 between AkAES and KbAES to 0.218 between KaAES and KvAES. Low levels of genetic differentiation were also observed between AkAES and KRIRG (*F_ST_* = 0.018), as well as between KRIAPG and KRIRG (*F_ST_* = 0.047). In contrast, the highest pairwise differentiation was observed for KaAES, which showed *F_ST_* values of 0.217 with KRIAPG, 0.190 with KRIRG, and 0.218 with KvAES. Relatively high differentiation was also detected between KRIRG and KvAES (*F_ST_* = 0.207) and between KRIAPG and KvAES (*F_ST_* = 0.176). Detailed results of AMOVA and *F_ST_* are provided in File S2.

### 2.3. Population Structure Inferred from Genome-Wide SNP Markers

**Figure 3:**
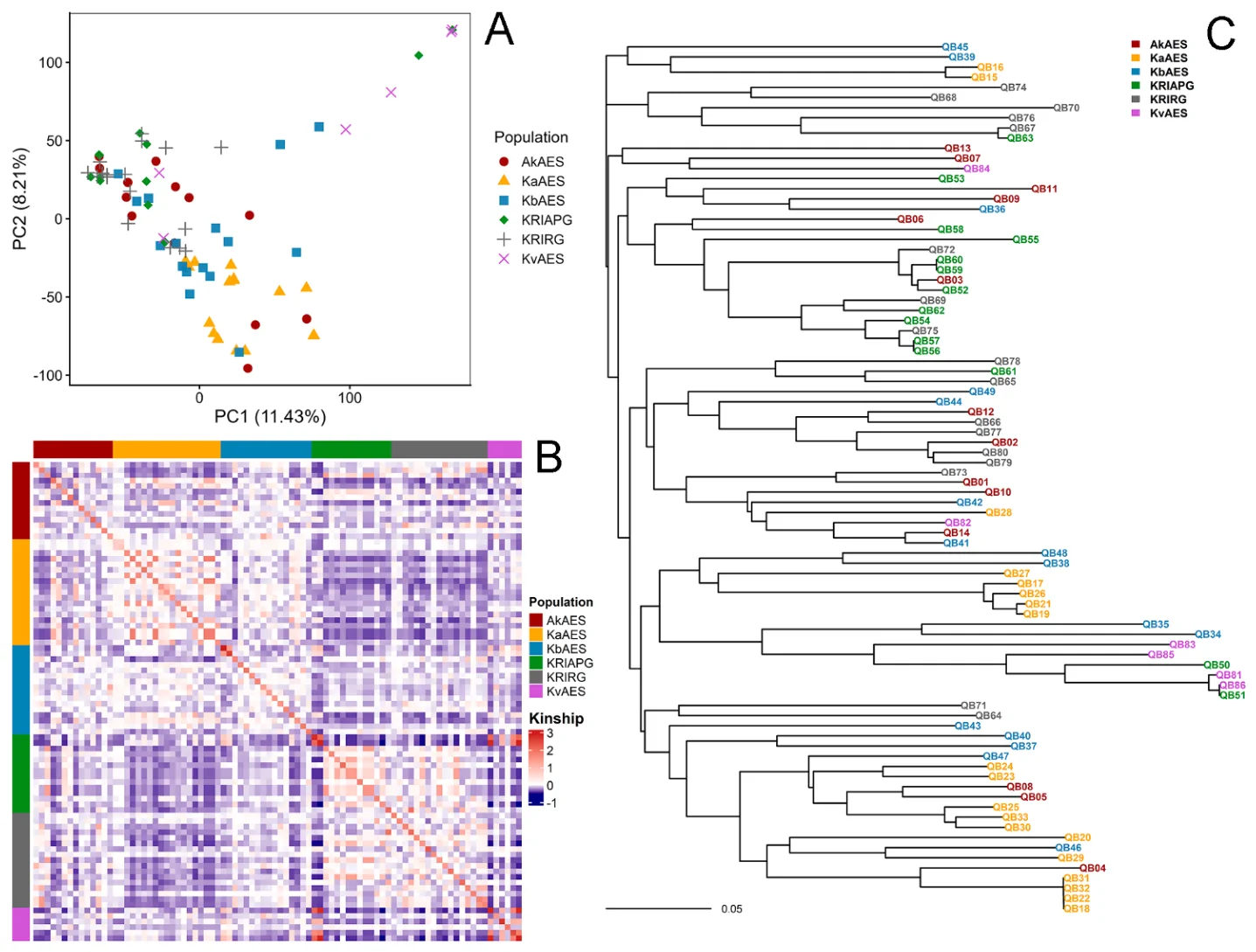
Population genetic structure and relationships among the studied barley accessions. (**A**) Principal component analysis (PCA) plot. (**B**) Heatmap of the pairwise genomic kinship matrix calculated using the VanRaden method. (**C**) Neighbor-joining (NJ) phylogenetic tree constructed from the Identity-by-state (IBS) genetic distance matrix. All resolved nodes received bootstrap support greater than 90%.

**Figure 4:**
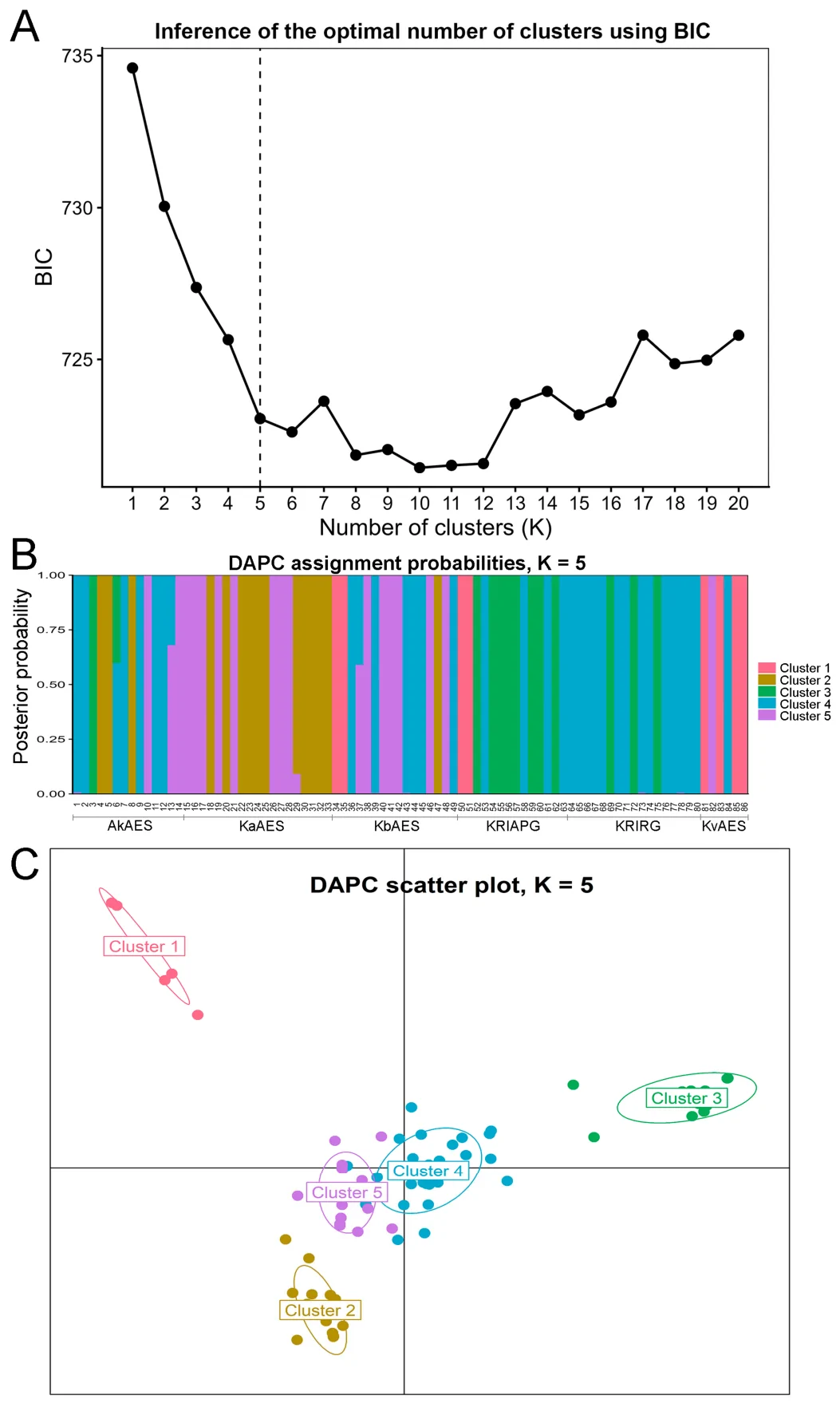
Discriminant Analysis of Principal Components (DAPC) of the 86 barley accessions. (**A**) Inference of the optimal number of clusters. BIC—Bayesian information criterion. (**B**) DAPC structure and assignment probabilities of the individual accessions at *K* = 5. (**C**) DAPC scatter plot mapping the genetic variation among the five clusters along the first two discriminant axes.

Principal component analysis (PCA), genomic kinship analysis, neighbor-joining (NJ) phylogenetic reconstruction (Figure 3), and discriminant analysis of principal components (DAPC) (Figure 4) were performed to investigate the population structure of the 86 barley accessions.

The first two principal components explained 11.43% and 8.21% of the total genetic variation, respectively (Figure 3A). Accessions originating from different breeding organizations were broadly intermixed on the PCA plot, and no distinct separation according to breeding origin was observed. Overall, substantial overlap occurred among breeding groups, although several accessions occupied more peripheral positions along the principal components. The genomic kinship heatmap revealed considerable variation in pairwise relatedness among accessions, with only limited correspondence between genomic relatedness and breeding origin (Figure 3B). Although several breeding groups contained moderately related accessions, highly related genotypes were also observed across different breeding organizations, and no clearly separated kinship blocks corresponding to breeding origin were detected. Similarly, the NJ tree did not recover monophyletic clusters corresponding to breeding organizations (Figure 3C). Accessions from different breeding programs were distributed across the phylogenetic tree, and most major branches contained genotypes originating from multiple breeding groups.

To further investigate the underlying genetic structure, DAPC was performed. The Bayesian Information Criterion (BIC) decreased rapidly with increasing numbers of inferred clusters, with the rate of decline becoming markedly less pronounced beyond *K* = 5 (Figure 4A). Accordingly, the optimal number of genetic clusters was determined as *K* = 5 using the elbow criterion rather than the minimum BIC value.

The DAPC analysis identified five well-defined genetic clusters, with most accessions assigned to a single cluster with high membership probabilities (Figure 4B). DAPC cluster membership was significantly associated with breeding organization (*χ^2^* = 106.38, df = 20, Monte Carlo *p* = 1 × 10^–5^). The bias-corrected Cramér’s *V* was 0.513 (bootstrap 95% CI: 0.456–0.650), indicating substantial differentiation among the represented breeding programmes. Standardized residuals revealed overrepresentation of KvAES in Cluster 1, KaAES in Cluster 2, KRIAPG in Cluster 3, and KRIRG in Cluster 4, whereas Cluster 5 was enriched in both KaAES and KbAES accessions. However, several clusters, particularly Cluster 4, included accessions from multiple organizations, demonstrating that breeding-origin differentiation was substantial but incomplete (Table S4).

The DAPC scatter plot further demonstrated clear genetic differentiation among the five inferred clusters, with limited overlap between Clusters 4 and 5 (Figure 4C). Comprehensive results of PCA, genomic kinship analysis, identity-by-state (IBS) analysis, NJ phylogenetic reconstruction, DAPC, and association between breeding organization and SNP-derived DAPC cluster membership are presented in File S3.

### 2.4. Spatial Distribution of Genetic Clusters

**Figure 5:**
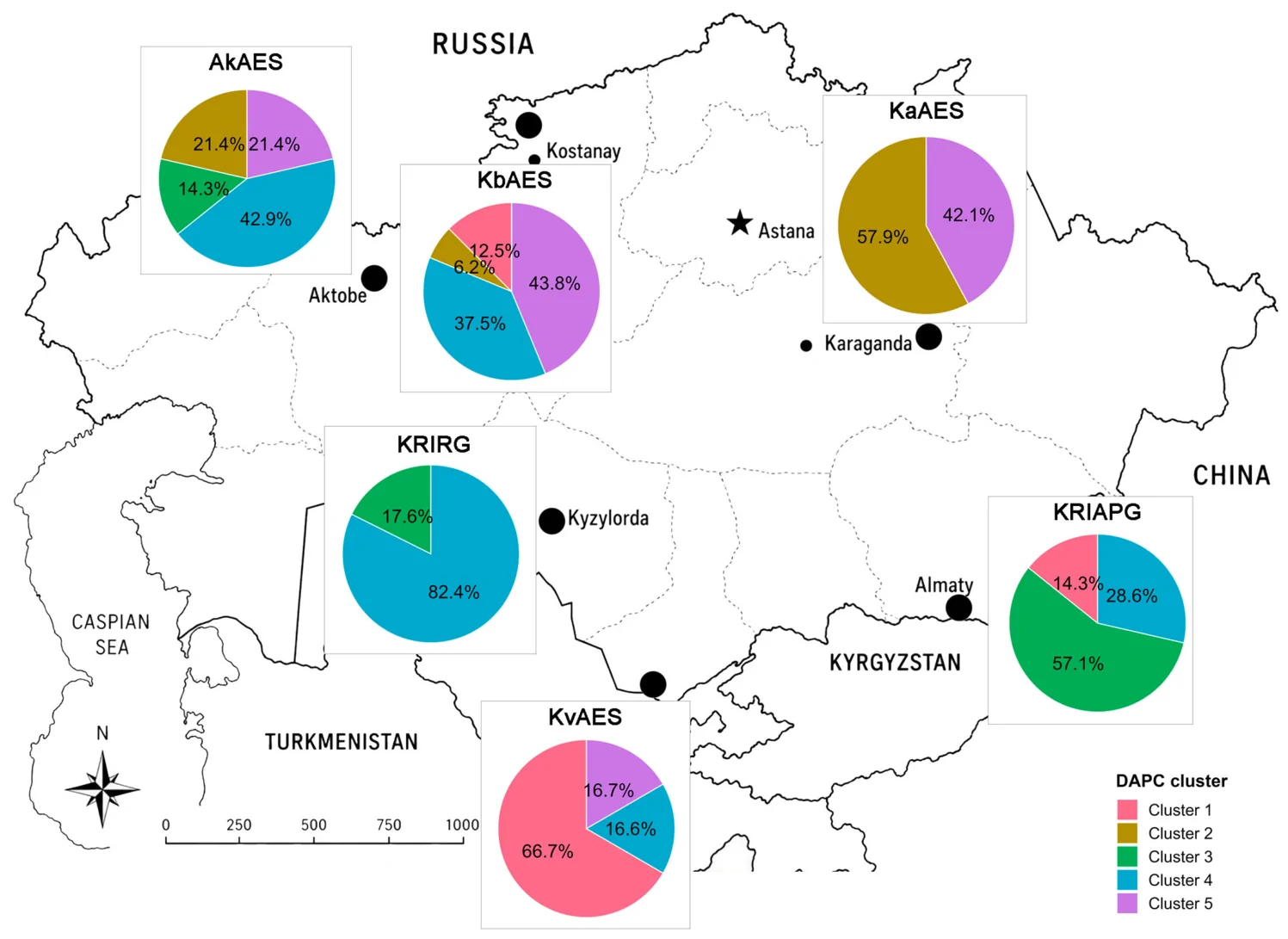
Discriminant Analysis of Principal Components (DAPC) genetic clustering framework of barley groups across the six breeding organizations in Kazakhstan. AkAES—Aktobe Agricultural Experimental Station, KbAES—Karabalyk Agricultural Experimental Station, KaAES—Karaganda Agricultural Experimental Station, KRIAPG—Kazakh Research Institute of Agriculture and Plant Growing, KRIRG—Kazakh Research Institute of Rice Growing, KvAES—Krasnovodopad Agricultural Experimental Station.

The geographic distribution of DAPC clusters demonstrated that no breeding organization represented an isolated genetic entity. Instead, all breeding organizations contained mixtures of multiple genetic clusters, although their relative proportions differed markedly (Figure 5).

The inferred genetic clusters were unevenly distributed among breeding organizations, although all organizations comprised accessions assigned to more than one DAPC cluster. Each breeding organization was characterized by a dominant genetic cluster: Cluster 4 predominated in AkAES and KRIRG, Cluster 5 in KbAES, Cluster 3 in KRIAPG, Cluster 2 in KaAES, and Cluster 1 in KvAES. The proportion of the dominant cluster varied substantially among breeding organizations, ranging from 42.9% in AkAES to 82.4% in KRIRG.

The composition of genetic clusters also differed among breeding organizations. AkAES and KbAES exhibited the highest cluster diversity, each containing accessions assigned to four of the five inferred clusters. KRIAPG and KvAES each comprised three clusters, whereas KaAES and KRIRG comprised two clusters. Despite differences in the relative abundance of individual clusters, all five DAPC clusters were represented across the complete barley collection, and no breeding organization corresponded exclusively to a single genetic cluster.

### 2.5. Phenotypic Differentiation and Characterization of SNP-Derived Genetic Clusters

Because breeding origin showed substantial but incomplete correspondence with SNP-derived DAPC cluster membership, the phenotypic differentiation among the inferred genetic clusters was further investigated. Best linear unbiased predictors (BLUPs) were calculated for seven key agronomic traits using phenotypic data collected during the 2025 growing season across four breeding environments (KaAES, KbAES, KRIAPG, and KRIRG) (Table S1). The analyzed traits included heading time (HT), heading-to-maturity time (HMT), vegetation period (VP), plant height (PH), peduncle length (PL), spike length (SL), and number of kernels per spike (NKS).

Analysis of variance (ANOVA) revealed significant phenotypic differences among the five SNP-derived DAPC clusters for all seven agronomic traits (Table 4).

The strongest differentiation was observed for NKS (FDR *p*-value = 2.51 × 10^–10^), followed by HT and SL (FDR *p*-value = 4.26 × 10^–4^ for both), while the remaining traits also differed significantly among genetic clusters (FDR *p*-value ≤ 0.004). Pairwise differences among clusters were further evaluated using Tukey’s honestly significant difference (HSD) test (Figure S2).

**Figure 6:**
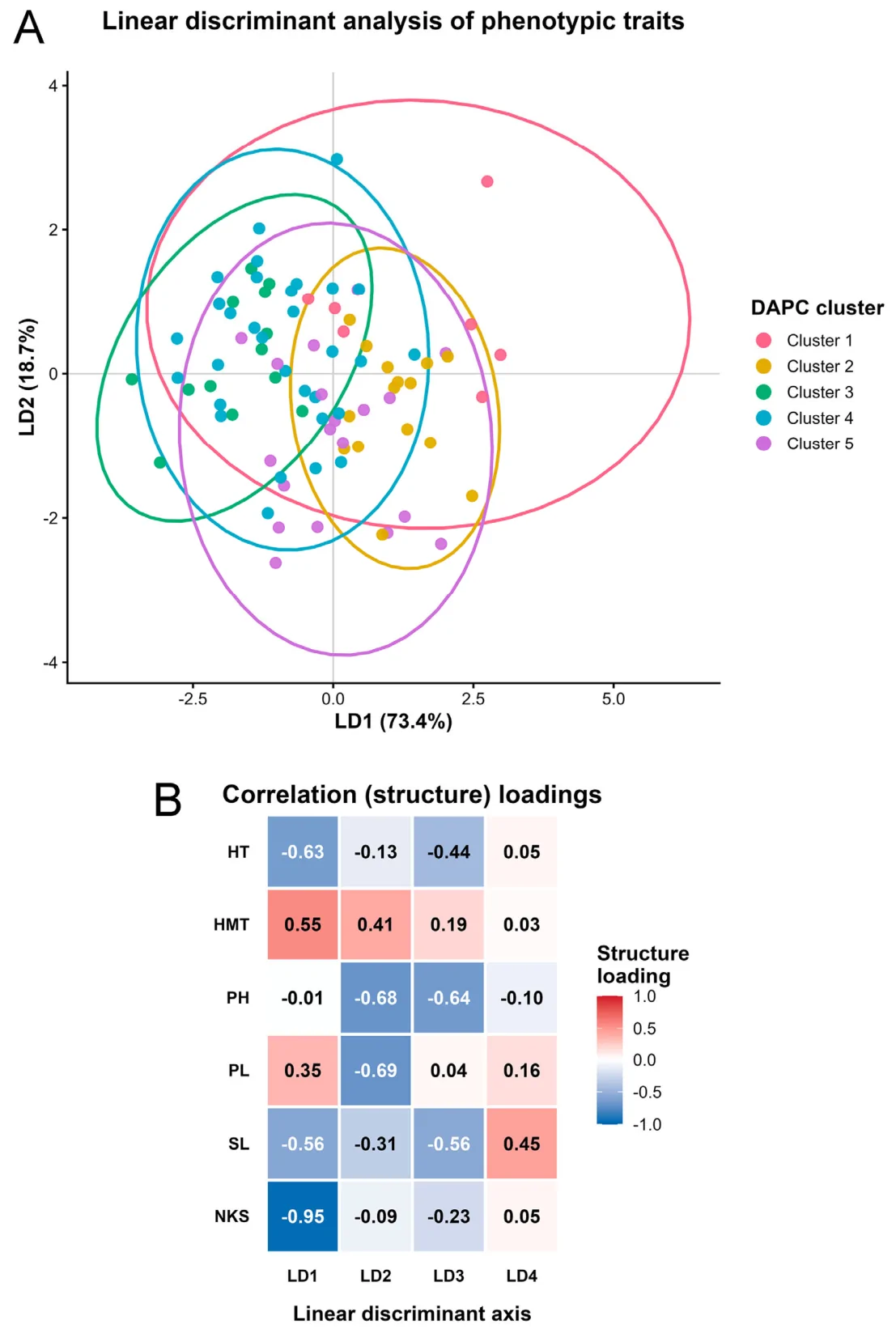
Phenotypic differentiation among SNP-derived genetic clusters identified by discriminant analysis of principal components (DAPC). (**A**) Linear discriminant analysis (LDA) based on six standardized genotype BLUPs. Ellipses represent 95% confidence regions for the five predefined DAPC clusters. (**B**) Correlation structure loadings between the six standardized phenotypic traits and the four linear discriminant functions. HT—heading time; HMT—heading-to-maturity time; PH—plant height; PL—peduncle length; SL—spike length; NKS—number of kernels per spike.

Pearson correlation analysis of genotype BLUPs revealed strong associations among the phenological traits, including a negative correlation between HT and HMT (*r* = −0.85) and a positive correlation between HT and VP (*r* = 0.74), confirming their substantial non-independence (Figure S3). The reduced six-trait LDA, in which VP was excluded to avoid near-collinearity with HT and HMT, provided partial separation of the five SNP-derived genetic clusters, although considerable overlap remained (Figure 6A).

LD1 and LD2 accounted for 73.4% and 18.7% of the total discrimination, respectively, explaining 92.1% cumulatively. Complete results of the LDA and LOOCV analyses, including standardized discriminant coefficients, structure loadings, accession-level predictions, the confusion matrix, per-cluster classification performance, and permutation test results, are provided in File S4. Structure loadings indicated that LD1 was most strongly associated with NKS (*r* = −0.95), followed by HT (*r* = −0.63), SL (*r* = −0.56), and HMT (*r* = 0.55), whereas LD2 was primarily associated with PL (*r* = −0.69) and PH (*r* = −0.68) (Figure 6B).

**Figure 7:**
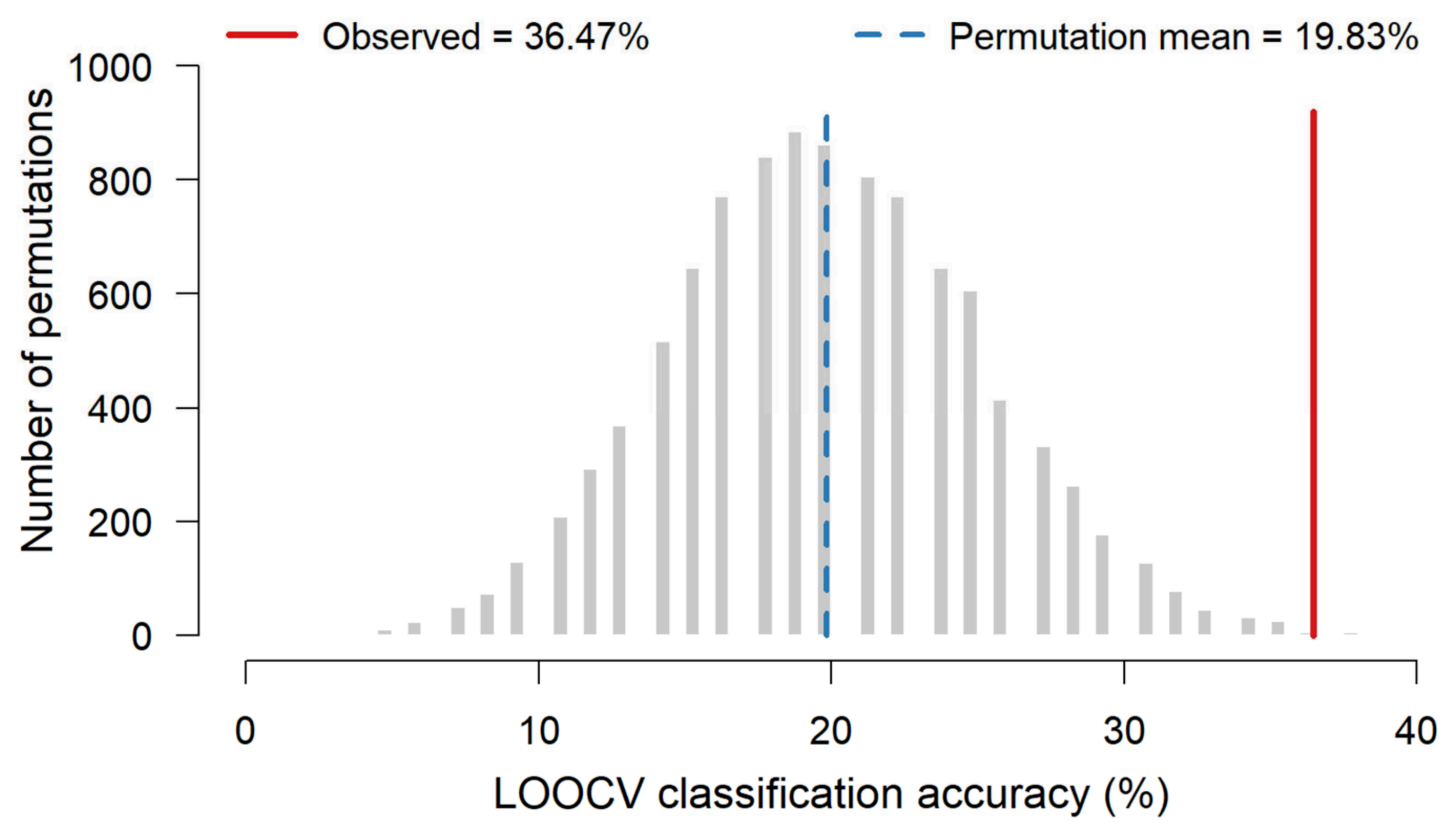
Permutation-based evaluation of leave-one-out cross-validation (LOOCV) accuracy for the six-trait linear discriminant analysis (LDA). Classification accuracy was obtained from 10,000 random permutations of DAPC cluster labels.

Leave-one-out cross-validation (LOOCV) correctly classified 31 of 85 accessions, corresponding to an overall accuracy of 36.47%. Across 10,000 permutations of cluster labels preserving the original cluster sizes, the mean expected accuracy was 19.83%, substantially lower than the observed value (Figure 7).

Cluster-specific assignment success was 4/7 (57.14%) for Cluster 1, 10/15 (66.67%) for Cluster 2, 6/13 (46.15%) for Cluster 3, 6/31 (19.35%) for Cluster 4, and 5/19 (26.32%) for Cluster 5. Although the overall accuracy equaled the majority-class baseline of 36.47%, it exceeded both the nominal equal-prior chance level of 20.00% and the permutation expectation (mean = 19.83%; 95% interval: 9.41–30.59%; permutation *p* = 0.0015). The observed balanced accuracy was 43.13% and also exceeded its permutation expectation (mean = 19.89%; 95% interval: 9.30–31.44%; permutation *p* < 0.001). Nevertheless, the absolute classification accuracy remained modest, consistent with considerable phenotypic overlap among the genetic clusters. Thus, phenotypic differentiation among the genetic clusters reflected a combination of yield-component, phenological, and plant architectural traits rather than a predominant contribution of phenology alone.

**Figure 8:**
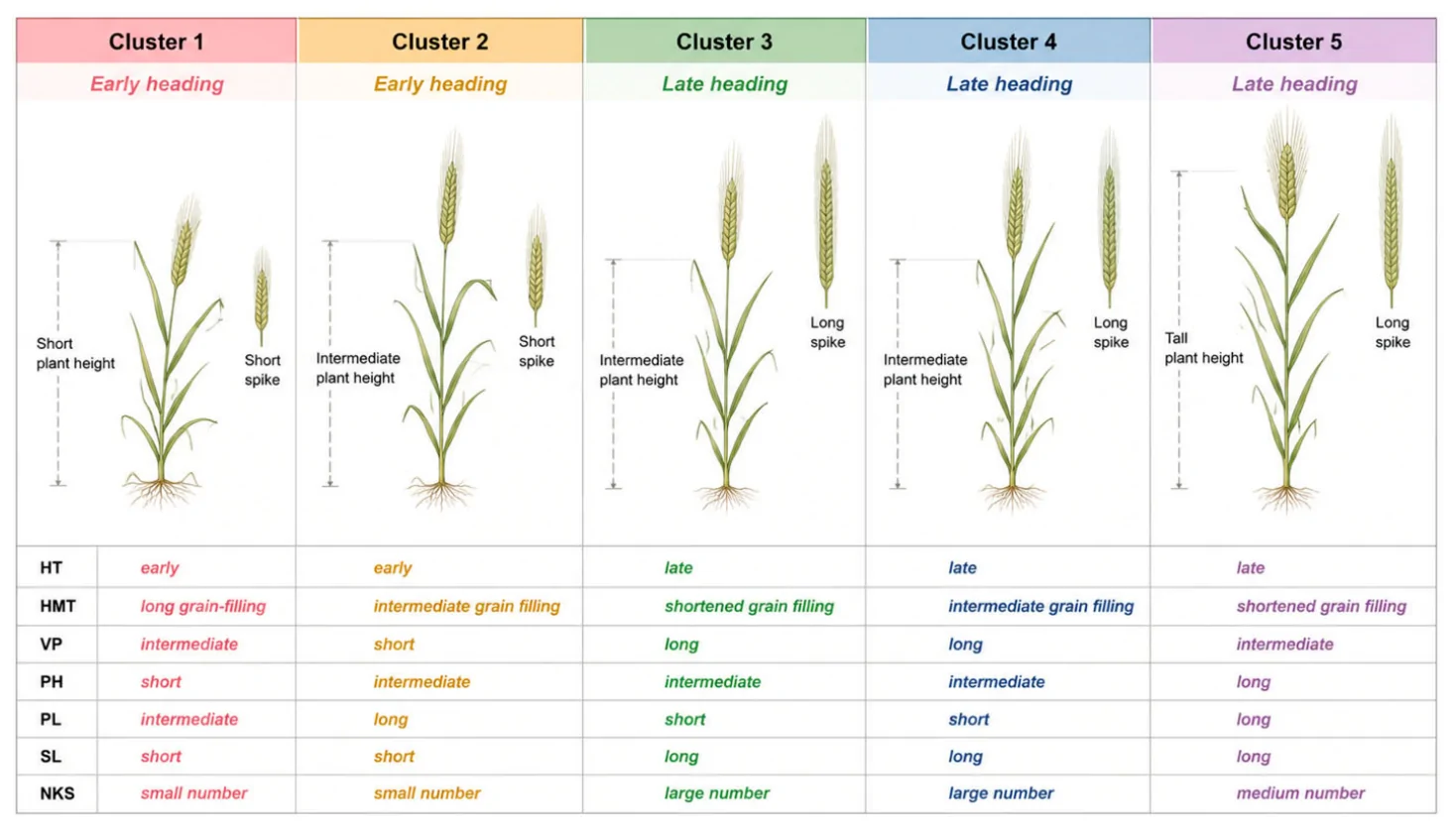
Cluster-associated phenotypic profiles of the five SNP-derived DAPC genetic clusters of barley. HT—heading time, HMT—heading-to-maturity time, VP—vegetation period, PH—plant height, PL—peduncle length, SL—spike length, NKS—number of kernels per spike.

Based on the significant phenotypic differences among DAPC clusters, five cluster-associated phenotypic profiles were summarized (Figure 8).

Cluster 1 represented an early-heading profile characterized by a prolonged grain-filling period, relatively short plants, short spikes, and a low number of kernels per spike. Cluster 2 was also early-heading but differed in a shorter vegetation period, an intermediate grain-filling duration, longer peduncles, and short spikes with a low kernel number. Cluster 3 comprised late-heading genotypes with a long vegetation period, shortened grain filling, long spikes, and a high number of kernels per spike. Cluster 4 also represented a late-heading profile but was distinguished from Cluster 3 by an intermediate grain-filling period while retaining long spikes and a high kernel number. Cluster 5 was characterized by late heading, an intermediate vegetation period, shortened grain filling, tall plants with long peduncles and spikes, and an intermediate number of kernels per spike. Together, these five profiles summarize the characteristic phenotypic profiles associated with each SNP-derived DAPC genetic cluster.

## 3. Discussion

### 3.1. The Analyzed Barley Breeding Panel Retains Moderate Genetic Diversity

Genome-wide SNP analysis demonstrated that the panel of two-row spring barley breeding materials retained moderate genetic diversity despite decades of directional selection. Expected heterozygosity (*H_e_* = 0.346 ± 0.169), polymorphism information content (PIC = 0.278 ± 0.127), and Shannon’s information index (0.752 ± 0.333) (Table 1) were comparable to those reported for other cultivated barley collections [7,14,23,29,30,31], indicating that appreciable genome-wide variation remained available within the analyzed breeding material. The uniformly high fixation indices (*F_IS_* = 0.996–0.999) together with the very low residual heterozygosity (*H_o_* = 0.0004) (Table 1) were expected for cultivated barley because of its predominantly self-pollinating reproductive system and the extensive inbreeding associated with elite cultivar development [32].

The diversity estimates obtained here closely agree with those reported previously for Kazakh barley germplasm. Using 5636 polymorphic SNPs to characterize 94 accessions, Almerekova et al. (2021) [23] reported *H_e_* values ranging from 0.131 to 0.337 and Shannon’s information index between 0.224 and 0.501. Our within-population estimates (*H_e_* = 0.272–0.328; Shannon’s index = 0.586–0.709, Table 1) were slightly higher but remained within a comparable range, possibly reflecting differences in marker density and SNP filtering. Importantly, both studies consistently indicate that the panel of two-row spring barley breeding materials has maintained substantial genetic variation despite long-term selection.

Similar patterns have been reported for cultivated barley worldwide. Analysis of the USDA Barley Core Collection, comprising more than 2400 accessions from over 100 countries, demonstrated that substantial allelic diversity is retained within cultivated barley despite intensive breeding, providing valuable genetic resources for genome-wide association studies and crop improvement [7]. Likewise, Brbaklić et al. (2021) [16] reported that the progressive introduction of novel parental material increased genetic diversity across successive breeding periods in Serbian barley. Studies of barley landraces have generally reported even higher allelic richness and genetic diversity than elite germplasm, reflecting their broader evolutionary histories and the weaker artificial selection acting on these traditional populations. For example, Pasam et al. (2014) [29] observed gene diversity (*H_e_*) values of 0.57–0.61 and PIC values of 0.52–0.56 in large collections of cultivated barley landraces genotyped with SSR markers, considerably exceeding those typically observed in modern breeding materials. Nevertheless, quantitative comparisons among studies should be interpreted cautiously because diversity estimates were generated using different marker systems (SSR vs. SNP), whose contrasting allelic resolution inevitably influences the magnitude of diversity indices.

This interpretation is further supported by the observed partitioning of genetic variation. Although AMOVA detected significant differentiation among organizations (19.16%), the vast majority of molecular variation (80.84%) remained within breeding groups (Table 3), indicating that individual organizations maintain considerable genetic diversity. The MAF-free reanalysis showed that 95.61% of the observed allele states at polymorphic loci were shared by at least two breeding organizations, supporting the presence of a broad common allelic pool (Table 2). Nevertheless, private-allele richness was strongly heterogeneous and remained highest in KbAES after sample-size standardization, indicating incomplete differentiation and organization-specific enrichment within this shared pool. Because approximately two-thirds of the detected private alleles were represented by a single accession, these rare variants should be interpreted cautiously and independently validated. Collectively, these findings indicated that the six represented breeding programs maintained considerable genetic diversity while sharing much of their allelic variation.

### 3.2. Population Structure Reflects Substantial but Incomplete Differentiation Among Breeding Programs

A central objective of the present study was to determine whether the analyzed barley panel is genetically structured according to breeding organization. Collectively, the complementary analyses indicated substantial but incomplete differentiation associated with breeding organization. Although accessions from different organizations overlapped in the PCA, kinship, and NJ analyses, the uneven distribution of breeding groups among DAPC clusters suggested that breeding origin contributed meaningfully to the observed genomic structure without producing complete genetic separation.

DAPC further refined this interpretation by identifying five genetic clusters (Figure 4A) and demonstrating broad cross-organization representation of DAPC clusters. Each breeding organization possessed a predominant genetic cluster, yet each organization contained accessions assigned to at least two clusters, and the breeding groups with the broadest DAPC-cluster representation (AkAES and KbAES) contained four of the five inferred clusters (Figure 5). Conversely, none of the inferred genetic clusters was confined to a single breeding organization, further supporting the extensive sharing of genetic variation among programs. This pattern contrasts with numerous previous studies in barley, where population structure reflected geographic origin or breeding history. For example, Comadran et al. (2009) [14] identified five major genetic groups corresponding to key ancestral lineages and regions of origin, while Zhou et al. (2012) [20] identified that most subpopulations were strongly represented by lines from specific US breeding programs. The population structure identified by Bernád et al. (2024) [33] was associated with the accession’s year of release.

The formal association test demonstrated substantial differentiation among breeding programs, with DAPC clusters enriched for accessions from particular organizations. Nevertheless, the presence of accessions from multiple organizations within several clusters, especially the broadly represented Cluster 4, indicates incomplete differentiation and supports the existence of a shared breeding core, potentially reflecting historical germplasm exchange alongside program-specific selection.

This combination of program-specific differentiation and cross-program genetic sharing was likely explained by the composition and breeding history of the analyzed germplasm. Unlike globally diverse barley collections, the present panel consisted exclusively of two-row hulled spring barley developed within a single national system. Consequently, the extensive admixture among organizations most likely reflected the long-standing exchange of elite cultivars, advanced breeding lines, and parental germplasm among Kazakh breeding institutions since the Soviet period, resulting in a largely shared breeding pool among the six represented organizations. The population differentiation statistics further support this interpretation. Although AMOVA revealed significant differentiation among organizations, only 19.16% of the total molecular variation was attributable to differences among breeding groups, whereas 80.84% remained within them (Table 3). Likewise, pairwise weighted *F_ST_* values ranged from 0.007 between AkAES and KbAES to 0.218 between KaAES and KvAES (Figure 2), indicating heterogeneous but generally moderate differentiation rather than complete genetic isolation. Thus, the observed genomic structure was consistent with both program-specific selection and historical germplasm sharing: breeding organization was significantly associated with DAPC cluster membership but did not produce complete genetic separation among programs.

### 3.3. Phenotypic Differentiation Among the Inferred Genetic Clusters

The incomplete correspondence between genetic clusters and breeding organization raises the question of which phenotypic characteristics accompany the observed genomic structure. One-way ANOVA detected significant differences among the five DAPC clusters for all seven evaluated traits, with the strongest univariate differentiation observed for NKS, followed by HT and SL (Table 4). Because VP showed substantial correlation with HT and was nearly dependent on HT and HMT (Figure S3), it was excluded from the multivariate LDA to reduce instability caused by collinearity. In the reduced six-trait model, LD1 was most strongly associated with NKS, followed by HT, SL, and HMT, whereas LD2 was primarily associated with PL and PH (Figure 6B). Thus, phenotypic differentiation among the inferred genetic clusters reflected a combination of yield-component, phenological, and plant architectural traits rather than a predominant contribution of phenology alone.

The relatively low LOOCV classification accuracy of 36.47% further indicated considerable phenotypic overlap among the genetic clusters. Consequently, the phenotypic traits evaluated here cannot reliably predict DAPC cluster membership when used independently of genomic data. Phenological traits nevertheless remained relevant components of the observed differentiation, consistent with the established importance of photoperiod response (*PPD*), vernalization (*VRN*), flowering time (*FT*), and other developmental pathways in barley adaptation [34,35,36,37,38,39]. However, the present results do not establish a direct causal relationship between these pathways and the genome-wide population structure.

Although the use of genotype BLUPs with environment fitted as a fixed effect reduced systematic variation among breeding stations, phenotypic evaluation was limited to a single growing season. Consequently, location-specific climatic conditions and genotype-by-environment interactions may still have influenced the estimated phenotypic differences, particularly for environmentally responsive phenological traits. Multi-location and multi-year trials will therefore be required to evaluate the temporal stability of the cluster-associated phenotypic profiles and the reproducibility of the observed LDA structure.

The cluster-level phenotypic profiles further illustrated the multidimensional nature of the observed differentiation. Clusters 3 and 4 were characterized by relatively late heading, extended vegetation periods, longer spikes, and higher numbers of kernels per spike, whereas Clusters 1 and 2 exhibited earlier heading and shorter vegetation periods (Figure 8). Cluster 5 combined late heading with comparatively tall plants and long peduncles, representing a distinct architectural profile. These profiles reflected distinct combinations of developmental and architectural traits, although their unequal frequencies among breeding organizations were consistent with the significant but incomplete origin–cluster association. A similar relationship between population structure and phenology has been reported in previous studies of cultivated barley. Malysheva-Otto et al. (2006) [13] demonstrated that genetic structure among 953 cultivated barley accessions was primarily associated with growth habit and row type rather than breeding institution, highlighting the importance of developmental traits as major determinants of genomic differentiation. Even if association mapping panels are selected within one of these phenotypic classes, sub-structure is still likely to be present due to other factors such as geographic origin and related pedigree [40]. Although the present panel was restricted to two-row spring barley, thereby controlling for two major determinants of barley population structure, significant variation remained in phenology, plant architecture, and yield components. The reduced LDA demonstrated that no single functional group of traits independently explained the inferred genetic clusters. Moreover, the limited LOOCV accuracy indicates that these phenotypic associations should be interpreted at the group level and should not be used alone to assign individual accessions to genetic clusters.

These findings also have important practical implications for barley improvement. First, breeding organization alone appears to be an insufficient criterion for selecting genetically diverse parental combinations because substantial genetic variation is shared among breeding programs. The inferred genetic clusters and their associated phenotypic profiles may complement genomic information during parent selection, although phenotypic profiles alone should not be used to infer cluster membership because of the considerable overlap observed among clusters. Several limitations should nevertheless be acknowledged. The analyzed panel was not intended to represent the complete contemporary barley gene pool of Kazakhstan, as it included both cultivars and advanced breeding lines from six participating organizations but did not encompass all officially approved domestic and foreign cultivars or all national breeding centers. Therefore, the observed population structure, phenotypic differentiation, and proposed breeding applications should be interpreted specifically within the examined two-row spring barley collections. A comprehensive national assessment will require broader sampling of registered cultivars, foreign germplasm, additional breeding programs, and other barley types. The present study included only two-row spring barley breeding germplasm from Kazakhstan and therefore does not capture the broader diversity represented by winter barley, six-row barley, landraces, or wild relatives. The phenotypic interpretation of the inferred genetic structure is based on group-level associations rather than direct analyses of causal loci, and validation through multi-year trials, functional markers, and genome-wide association studies will be required. Despite these limitations, the present results provide a practical framework for parent selection, future GWAS and genomic selection studies, and the management of genetic diversity in Kazakh barley breeding programs.

## 4. Materials and Methods

### 4.1. Plant Germplasm

A panel of 86 accessions representing two-row spring barley breeding material of Kazakhstan was used in this study. The collection comprised cultivars and advanced breeding lines originating from six barley breeding organizations in Kazakhstan: Aktobe Agricultural Experimental Station (AkAES, 50.3215° N, 57.1560° E, *n* = 14), Karaganda Agricultural Experimental Station (KaAES, 50.1792° N, 72.7412° E, *n* = 19), Karabalyk Agricultural Experimental Station (KbAES, 53.8516° N, 62.1018° E, *n* = 16), Kazakh Research Institute of Agriculture and Plant Growing (KRIAPG, 43.2227° N, 76.6832° E, *n* = 14), Kazakh Research Institute of Rice Growing (KRIRG, 44.8309° N, 65.5114° E, *n* = 17), and Krasnovodopad Agricultural Experimental Station (KvAES, 41.4538° N, 69.1756° E, *n* = 6). Based on information provided by the originating breeding organizations and verification against the State Register of Breeding Achievements of the Republic of Kazakhstan as of August 2026 [41], the panel comprised 13 cultivars officially registered for use in Kazakhstan, 33 cultivars not registered for use in Kazakhstan, and 40 advanced breeding lines. The designation “cultivar” refers to the breeding status of a named variety, whereas its registration status indicates whether it was included in the current State Register of Kazakhstan.

Accessions were grouped by breeding organization and used as predefined populations for analyses of genetic diversity and population differentiation. Detailed information for each accession, including its identifier, breeding organization, material category, and official registration status in Kazakhstan, is provided in Table S2.

### 4.2. SNP Genotyping and Quality Control

Genomic DNA was extracted from young barley seedlings using the Plant Pro Kit (QIAGEN, Hilden, Germany) according to the manufacturer’s instructions. Genotyping was performed by TraitGenetics GmbH (Gatersleben, Germany) using the Illumina Infinium iSelect 50K Barley SNP Array, comprising 44,040 SNP markers. Genotypes were coded as 0, 1, and 2 according to the number of alternative alleles. Quality control, downstream data preparation, subsequent SNP density, and LD analyses were performed using a custom R-based bioinformatics pipeline developed for this study and implemented as a fully reproducible workflow. The complete source code and documentation are publicly available through GitHub [42] and archived in Zenodo [42]. The pipeline automatically performed data validation, quality control, marker harmonization, and preparation of all datasets required for subsequent analyses.

Quality filtering was applied to both SNP markers and accessions. SNPs with a MAF < 0.05, SNP call rate < 0.90, SNP heterozygosity > 0.10, as well as accessions with call rate < 0.90 or heterozygosity > 0.10 were excluded from further analyses. Physical positions of SNP markers were assigned according to the Morex v3 barley reference genome [43]. Following quality filtering, 29,920 high-quality SNPs were retained for genetic diversity and population structure analyses; of these, 28,357 had known physical positions and were used for chromosome-based analyses, including SNP density and LD.

### 4.3. Genetic Diversity and Population Structure Analyses

The pipeline integrated analyses implemented in R, PLINK v1.9 [44], and VCFtools [45] and automatically generated all summary statistics, tables, and publication-quality figures used in this study. Genetic diversity was assessed separately for each breeding organization by calculating the observed heterozygosity (*H_o_*), expected heterozygosity (*H_e_*), PIC, Shannon’s information index, inbreeding coefficient (*F_IS_*), and PPL.

For the private-allele analysis, the original 44,040-SNP dataset was filtered independently without applying a minor-allele-frequency threshold. Accessions were required to have a call rate ≥ 0.90 and heterozygosity ≤ 0.10, while SNPs were retained when their overall call rate was ≥0.90 and their call rate within each breeding organization was ≥0.80. A nucleotide allele was classified as private when it was observed exclusively in one breeding organization. Private alleles represented by at least two accessions were additionally summarized as more strongly supported variants. To account for unequal sample sizes, the recovery of full-panel-confirmed private alleles was rarefied to six accessions per organization using 10,000 random subsamples.

Genetic differentiation among breeding organizations was evaluated using AMOVA based on Nei’s genetic distance with 999 permutations and pairwise weighted fixation indices (*F_ST_*) estimated using VCFtools.

Population structure was investigated using principal component analysis (PCA), a genomic kinship matrix calculated according to the VanRaden method, a NJ tree constructed from pairwise identity-by-state (IBS) genetic distances, and DAPC [46]. Branch support was assessed using 1000 bootstrap replicates by resampling SNP loci with replacement and reconstructing the IBS-based NJ tree for each replicate. Prior to PCA and DAPC, missing genotypes were imputed using the marker mean. The optimal number of genetic clusters in DAPC was determined using BIC and the elbow method. The final model retained the first 20 principal components for discriminant analysis.

The association between breeding organization and DAPC cluster membership was evaluated using a contingency-table Pearson chi-square test. Statistical significance was estimated using 99,999 Monte Carlo replicates with fixed marginal totals. Association strength was quantified using bias-corrected Cramér’s V, with a 95% confidence interval obtained from 10,000 stratified bootstrap replicates; standardized Pearson residuals were used to identify organization–cluster combinations responsible for the association.

All of the above-mentioned genetic diversity and population structure analyses were performed using the same custom R-based bioinformatics pipeline developed for this study [42]. The geographic distribution of inferred genetic clusters across breeding organizations was visualized on a map using proportional pie charts based on DAPC assignments.

### 4.4. Phenotypic Evaluation and Multivariate Statistical Analyses

Phenotypic evaluation was conducted under field conditions during the 2025 growing season at four breeding stations located in different agroecological regions of Kazakhstan (KaAES, KbAES, KRIAPG, KRIRG). One accession, QB34, was not phenotyped. Seven agronomic traits were recorded, including heading time (HT), heading-to-maturity time (HMT), vegetation period (VP), plant height (PH), peduncle length (PL), spike length (SL), and number of kernels per spike (NKS). To account for environmental variation among field sites, BLUPs were calculated for each accession and trait using a linear mixed-effects model with environment treated as a fixed effect and genotype as a random effect. The resulting BLUP values were used in all subsequent phenotypic analyses.

One-way analysis of variance (ANOVA) was used to evaluate phenotypic differences among the five SNP-derived DAPC clusters. Model assumptions were assessed using the Shapiro–Wilk test for normality of residuals and Levene’s test for homogeneity of variances. Raw *p*-values from the seven one-way ANOVAs were adjusted for multiple testing using the Benjamini–Hochberg false discovery rate (FDR) procedure. Pairwise comparisons among DAPC clusters were subsequently performed using Tukey’s honestly significant difference (HSD) test. Pearson correlations were calculated among genotype BLUPs for the seven phenotypic traits, and the corresponding *p*-values were adjusted using the Benjamini–Hochberg FDR procedure.

Linear discriminant analysis (LDA) was performed using standardized BLUPs for six traits (HT, HMT, PH, PL, SL, and NKS), five predefined DAPC groups, and equal priors. Standardized coefficients and Pearson correlation structure loadings were calculated. LOOCV provided overall, balanced, and per-cluster performance, which was compared with a null distribution from 10,000 label permutations preserving cluster sizes using add-one-corrected Monte Carlo *p*-values. These analyses were intended to characterize group-level phenotypic differentiation among the SNP-derived genetic clusters and to assess the relative contributions of the evaluated agronomic traits.

All analyses were performed in R using the packages *lme4*, *emmeans*, *car*, *MASS*, *caret*, *dplyr*, *tidyr*, and *multcompView*.

## 5. Conclusions

Despite decades of intensive selection, the analyzed panel of two-row spring barley breeding materials retained moderate genetic diversity, with most molecular variation occurring within rather than among breeding organizations. Although PCA, genomic kinship, and neighbor-joining analyses revealed considerable overlap among organizations, DAPC cluster membership was significantly associated with breeding origin (Monte Carlo *P* < 0.001; bias-corrected Cramér’s *V* = 0.513), demonstrating substantial but incomplete differentiation among breeding programs within a shared breeding pool. All seven phenotypic traits differed significantly among the inferred genetic clusters. However, the six-trait LDA showed that this differentiation reflected a combination of yield-component, phenological, and plant architectural traits rather than a predominant contribution of phenology. The number of kernels per spike showed the strongest structural loading on LD1, while the LOOCV accuracy of 36.47% indicated substantial phenotypic overlap and limited ability to predict genetic cluster membership from phenotypic traits alone. The identified genomic clusters and their associated phenotypic profiles may support germplasm management and the selection of genetically divergent parents within the six represented collections. Future multi-year and multi-location phenotyping, followed by genome-wide association analysis and independent validation of candidate loci, will be required before individual molecular markers can be recommended for marker-assisted selection.

## Figures and Tables

**Table 1:** SNP genotyping quality and genetic diversity parameters of the two-row spring barley panel grouped by breeding organization (population).

Population	*N*	Call Rate (%)	MAF	*H_o_*	*H_e_*	PIC	Shannon	*F_IS_*	PPL (%)
AkAES	14	99.83 ± 1.41	0.23 ± 0.15	0.0005 ± 0.0076	0.309 ± 0.161	0.248 ± 0.120	0.670 ± 0.312	0.998 ± 0.036	88.52
KaAES	19	99.86 ± 1.40	0.20 ± 0.16	0.0003 ± 0.0064	0.272 ± 0.188	0.217 ± 0.143	0.586 ± 0.376	0.998 ± 0.034	77.62
KbAES	16	99.82 ± 1.32	0.24 ± 0.14	0.0005 ± 0.0070	0.328 ± 0.149	0.263 ± 0.109	0.709 ± 0.281	0.998 ± 0.027	93.14
KRIAPG	14	99.86 ± 1.27	0.20 ± 0.14	0.0004 ± 0.0066	0.280 ± 0.161	0.228 ± 0.121	0.617 ± 0.316	0.998 ± 0.032	86.68
KRIRG	17	99.89 ± 1.01	0.22 ± 0.15	0.0003 ± 0.0053	0.298 ± 0.168	0.239 ± 0.126	0.647 ± 0.327	0.998 ± 0.025	87.27
KvAES	6	99.70 ± 2.99	0.21 ± 0.16	0.0009 ± 0.0162	0.281 ± 0.187	0.224 ± 0.144	0.599 ± 0.384	0.996 ± 0.058	73.10
Total	86	99.84 ± 1.57	0.25 ± 0.15	0.0004 ± 0.0082	0.346 ± 0.169	0.278 ± 0.127	0.752 ± 0.333	0.999 ± 0.035	100.00

Values are presented as mean ± standard deviation (SD). *N*—number of accessions, MAF—minor allele frequency, *H_o_*—observed heterozygosity, *H_e_*_—_expected heterozygosity, PIC—polymorphism information content, *F_IS_*—inbreeding coefficient (fixation index within populations), PPL—percentage of polymorphic loci, AkAES—Aktobe Agricultural Experimental Station, KaAES—Karaganda Agricultural Experimental Station, KbAES—Karabalyk Agricultural Experimental Station, KRIAPG—Kazakh Research Institute of Agriculture and Plant Growing, KRIRG—Kazakh Research Institute of Rice Growing, KvAES—Krasnovodopad Agricultural Experimental Station.

**Table 2:** Distribution and sample-size-standardized richness of private SNP alleles among the six represented barley breeding organizations.

Breeding Organization	Number of Accessions	Total Private Alleles	Singleton Private Alleles	Private Alleles with ≥ 2 Carriers	Rarefied Private-Allele Richness, Mean (95% Interval)
KaAES	19	127	32	95	79.4 (42–111)
KbAES	16	1684	878	806	882.2 (309.3–1330)
KRIAPG	14	221	219	2	94.9 (3–205)
KRIRG	17	565	472	93	227.1 (26–502)
AkAES	14	280	238	42	131.5 (18–243)
KvAES	6	300	289	11	300.0 (300–300)

**Table 3:** Analysis of molecular variance (AMOVA) among the six Kazakh barley breeding groups of origin.

Source of Variation	Variance	Percent, %	*p*-Value
Among groups	0.01986	19.16	0.001
Within groups	0.08379	80.84	
Total	0.10365	100.00	

**Table 4:** Analysis of variance (ANOVA) for phenotypic traits among SNP-derived DAPC clusters.

Trait	Df	SS	MS	F-Value	*p*-Value	FDR *p*-Value
HT	4	50.891	12.723	6.540	1.32E–04	4.26 × 10^–4^
HMT	4	1.827	0.457	5.005	0.001	0.002
VP	4	7.316	1.829	4.330	0.003	0.003
PH	4	37.067	9.267	4.507	0.002	0.003
PL	4	18.995	4.749	4.651	0.002	0.003
SL	4	5.199	1.300	6.309	1.83E–04	4.26 × 10^–4^
NKS	4	138.627	34.657	19.358	3.59E–11	2.51 × 10^–10^

Df—degree of freedom, SS—sum of squares, MS—mean squares, FDR—false discovery rate, HT—heading time, HMT—heading-to-maturity time, VP—vegetation period, PH—plant height, PL—peduncle length, SL—spike length, NKS—number of kernels per spike.

## Data Availability

The genome-wide SNP genotyping dataset generated and analyzed in this study is publicly available in Zenodo: Genievskaya, Y.; Turuspekov, Y.; Abugalieva, S. Genome-Wide SNP Genotyping Dataset of 86 Modern Kazakh Barley Accessions [Dataset]. Zenodo, 2026. https://doi.org/10.5281/zenodo.21394007. The custom bioinformatics pipeline used for data processing and analysis is available in the GitHub repository: Genievskaya, Y. Barley Population Structure Pipeline. Available online: https://github.com/yuliya-genievskaya/Barley_Population_Structure_Pipeline (accessed on 19 July 2026), and has also been permanently archived in Zenodo: Genievskaya, Y. Barley Population Structure Pipeline, version 1.0.1 [Computer software]. Zenodo, 2026. https://doi.org/10.5281/zenodo.21263003. All other data supporting the findings of this study are included in the article and its Supplementary Materials.

## Supplementary Materials

The following supporting information can be downloaded at https://www.mdpi.com/article/10.3390/ijms27167466/s1.

## Abbreviations

The following abbreviations are used in this manuscript:

AMOVA	Analysis of molecular variance
ANOVA	Analysis of variance
BIC	Bayesian information criterion
BLUP	Best linear unbiased prediction
DAPC	Discriminant analysis of principal components
FDR	False discovery rate
LD	Linkage disequilibrium
LDA	Linear discriminant analysis
LOOCV	Leave-one-out cross-validation
NJ	Neighbor-joining
PCA	Principal component analysis
PIC	Polymorphism information content
PPL	Percentage of polymorphic loci
